# Load-Balanced Dynamic SFC Migration Based on Resource Demand Prediction

**DOI:** 10.3390/s24248046

**Published:** 2024-12-17

**Authors:** Tian Sun, Hefei Hu, Sirui Zhang

**Affiliations:** School of Information and Communication Engineering, Beijing University of Posts and Telecommunications, Beijing 100876, China; sunttian@bupt.edu.cn (T.S.); siruizhangtj01@gmail.com (S.Z.)

**Keywords:** network function virtualization, service function chaining, resource demand prediction, network load balancing, deep reinforcement learning

## Abstract

In network function virtualization, the resource demand of network services changes with network traffic. SFC migration has emerged as an effective technique for preserving the quality of service. However, one important problem that has not been addressed in prior studies is how to manage network load while maintaining service-level agreements for time-varying resource demands. Therefore, we propose the Resource Predictive Load Balancing SFC Migration (RP-LBM) algorithm in this paper. The algorithm uses CNN-AT-LSTM to predict VNF resource demands in advance, eliminating the delays associated with dynamic migrations and determining the optimal migration timing. It leverages the PPO algorithm’s perceptual capabilities in complex environments to develop SFC migration strategies and ensure network load balancing. Additionally, it reduces the number of subsequent migrations and minimizes the service interruption rate. The simulation results show that the service interruption rate of the RP-LBM algorithm is on average 27.3% lower than that of the passive migration method. The PPO-based migration algorithm has lower SFC migration times and service interruption rates compared to the DQN algorithm, ensuring service continuity with low migration costs.

## 1. Introduction

In traditional networks, deploying or updating the physical network requires specialized hardware to provide new services. This leads to high purchasing and maintenance costs. Additionally, the system cannot be easily customized or adjusted based on demand. Therefore, Network Function Virtualization (NFV) was developed. NFV aims to implement the functions of traditional network devices through software. It decouples network functions from dedicated hardware and runs them as software on general-purpose servers or in virtualized environments. This approach can significantly reduce operators’ costs and improve the flexibility and resilience of network service deployment. Network operators can use Service Function Chains (SFCs) to provide customized network services to users. SFC connects Virtualized Network Functions (VNFs). It not only supports the fine-grained and elastic delivery of services in the network but also allows for the modification of service functions and the migration of loads.

In NFV, operators use Service Function Chains to provide customized network services [1]. Network efficiency is closely related to the mapping of Virtualized Network Functions and the routing of SFCs. At the same time, as network slicing operates, the traffic arrival of each specific service fluctuates over time. This can lead to a mismatch between SFC resource demand and server resource availability, which negatively affects the quality of service (QoS) and resource utilization [2]. When a VNF layout and resource allocation strategy cannot meet the current network demands, the NFV Orchestrator (NFVO) reconfigures the SFC. This reconfiguration includes vertical scaling, horizontal scaling, and dynamic migration. Horizontal scaling makes full use of fragmented resources, but it is only possible when there are enough available resources on the server. Dynamic migration is a flexible solution during overload conditions, but it also incurs overhead.

Due to the dynamic changes in traffic, edge computing networks often experience overloaded nodes. This leads to increased computation delays and sometimes service interruptions. To maintain normal SFC services, SFC migration must be performed. Additionally, network load balancing should be ensured. Network load balancing allows the network system to adapt more easily to dynamic changes in traffic [3]. By introducing load balancing for time-varying network traffic, the network becomes more robust after VNF migration and promotes long-term resource optimization.

Based on the above background, we focus on the problem of SFC dynamic migration for network load balancing in edge computing environments where resources are scarce and highly dynamic. We use machine learning algorithms to predict VNF resource demands, which helps us anticipate changes in network node resource allocation. With the prediction results, we proactively determine the appropriate time for migration and identify the VNFs to migrate from overloaded nodes. We also use deep reinforcement learning algorithms to better understand complex environments and select target nodes for migration. This approach ensures network load balancing, reduces the number of future migrations, and minimizes the impact of unnecessary migrations.

In view of the above problems, the main contributions of this paper are as follows:

1. Considering the real SFC request scenario, we use a time-varying traffic dataset. We apply a CNN-AT-LSTM model to predict the short-term resource demands of VNFs. Using these predictions, we can proactively migrate VNFs in advance within a certain time frame.
2. Considering that dynamic changes in traffic may lead to frequent VNF migration and uneven use of network resources, we introduce a load-balancing model to maintain network stability.
3. In response to the multi-dimensionality and complexity of the VNF migration remapping problem caused by the dynamic network environment, we propose a DRL algorithm based on Proximal Policy Optimization (PPO) to enhance the effectiveness of VNF migration decisions.

## 2. Related Work

Within the framework of Network Function Virtualization, researchers from around the world have conducted many studies on the dynamic migration of Service Function Chains and have achieved significant results. One major challenge in dynamic migration is how to allocate resources for SFCs as their resource demands change and the duration that SFCs remain in the network is uncertain. Recent research typically takes one or more factors related to migration costs as optimization objectives and proposes corresponding migration decision algorithms.

For instance, the authors of [4] proposed a migration algorithm based on Deep Q-Networks (DQNs). Their goal was to minimize end-to-end latency and address service interruptions caused by overloaded nodes, link failures, and VNF instance failures. The authors of [5] introduced a framework that uses Convolutional Neural Networks (CNNs) and Artificial Neural Networks (ANNs) to determine the optimal migration paths for VNFs. Their aim was to minimize migration time and cost. This solution analyzes network conditions, workload patterns, and resource availability to achieve dynamic and efficient VNF relocation, which significantly improves network performance and reliability. Additionally, the authors of [6] presented a scalable cluster-based VNF migration algorithm. This algorithm is designed to minimize the total embedding cost while meeting the latency requirements between VNFs. It performs better than existing methods in terms of embedding costs and has a much shorter execution time compared to brute-force search methods.

However, the methods mentioned above only trigger migrations after changes occur in network traffic or after network node or link failures. These are forms of reactive migration and cannot proactively handle sudden changes in resource demand. Currently, to solve reactive migration, researchers are trying to predict resource demands in advance. In [7], the authors proposed a CAT-LSTM model to predict the resource demands of VNFs using SFC data. They improved accuracy by using aspect embedding and attention mechanisms. Similarly, the authors of [8] introduced a Graph Neural Network (GNN) model based on the graph features. This model accurately predicts VNF resource demands by identifying multidimensional dependencies within SFC graph structures. Although studies in [7,8] have made progress in prediction, they do not deeply explore how to effectively use these predictions to optimize resource allocation.

Moreover, existing research primarily focuses on optimizing objectives such as reducing latency and migration costs. However, merely minimizing energy consumption or overhead may lead to uneven utilization of network resources, and future dynamic traffic changes could result in frequent VNF migrations. Additionally, in edge computing networks where resources are relatively scarce and highly dynamic, greater emphasis should be placed on the distribution and utilization efficiency of resources to ensure network load balancing.

Current studies have demonstrated the advantages of ensuring network load balancing in enhancing network performance. For example, the authors of [9] proposed a load-balanced Virtual Network Embedding algorithm based on deep reinforcement learning for regional satellite networks. The algorithm divides the satellite network into multiple mapping regions and deploys Virtual Network Requests only in the region with the lowest load. It simultaneously considers topology dynamics and resource loads, effectively improving acceptance rates while also enhancing resource utilization. Similarly, the authors of [10] introduced a task scheduling algorithm considering load balancing issues in cloud computing environments. The proposed BCSV algorithm achieves better results in terms of completion time, load balancing, and resource utilization compared to previous algorithms, even in heterogeneous environments. This improvement enhances the overall performance of cloud computing networks.

In summary, we use high-precision predictions to anticipate SFC resource demands in advance and determine the appropriate timing for migration. We introduce a load-balancing model to ensure efficient use of resources, reduce the frequency of VNF migrations in the network, and avoid potential issues caused by uneven resource usage. Additionally, we design a deep reinforcement learning algorithm to proactively make migration decisions for SFCs on overloaded nodes, preventing the degradation or even interruption of network service quality.

## 3. System Model and Problem Description

In this section, we define the network model, describe the SFC and VNF, and then propose the VNF migration problem and the network load balancing model.

### 3.1. Network Model

**Figure 1:**
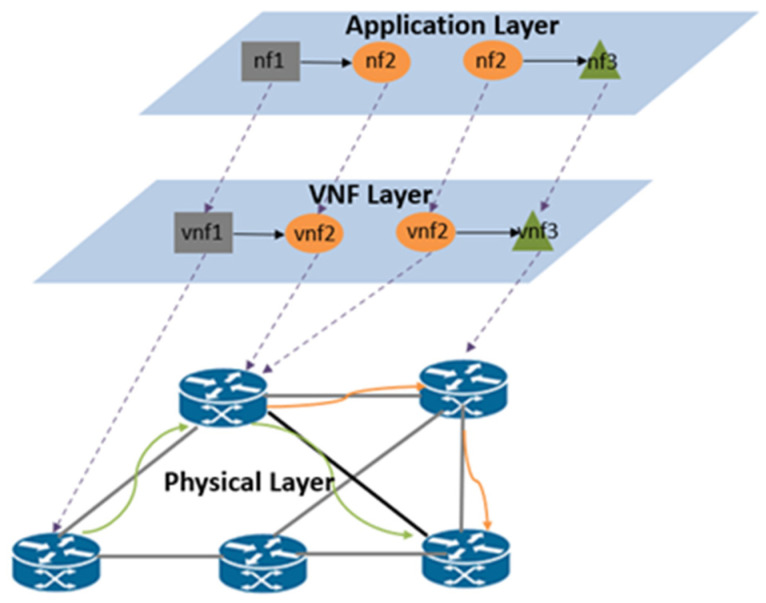
SFC mapping architecture.

We consider an NFV network architecture with three layers as depicted in [11]. As shown in Figure 1, physical nodes are connected by links in the physical layer. VNFs are instantiated on physical nodes that supply the necessary resources in the VNF layer. The various NF types that comprise the SFC are deployed on matching VNF types at the application layer.

The physical network is modeled as a fully connected undirected graph $G=\left(V^{s},E^{s}\right)$, where $V^{s}$ is the set of underlying nodes and $E^{s}$ is the set of physical links between nodes. The underlying nodes have a set of resource types to support VNFs, such as CPU, memory, and cores. Here, $C_{i}^{S}$ represents the maximum CPU resource capacity of the i-th node $v_{i}^{s}\in V^{S}$, where each unit of CPU resource represents the resources required to process a data packet. The memory resource capacity of the i-th node is $M_{i}^{S}$, represented in available megabytes. The underlying link directly connecting nodes $v_{i}^{s}$ and $v_{j}^{s}$ is represented as $e_{ij}^{S}\in E^{s}$, the physical link bandwidth is $BW_{ij}^{S}$, and the link delay is represented as $L_{ij}^{S}$.

### 3.2. SFC and VNF

The VNF type set is represented as F, and the CPU and memory resources consumed by the server to instantiate a VNF f∈F, denoted as $\;v^{vnf}$, are $c^{f}$ and $m^{f}$, respectively. A physical node can instantiate one or more types of VNFs, and each type of VNF can support the deployment of multiple virtual network function instances.

The SFC set is represented as S, and each request $sfc^{j}\in S$ can be represented as $sfc^{j}=\langle v_{j,in}^{s},v_{j,out}^{s},V_{j}^{NF},E_{j}^{NF},l_{j}\rangle$, where $v_{j,in}^{s}$ and $v_{j,out}^{s}$ represent the entrance and exit network elements, respectively; $V_{j}^{NF}$ represents a set of ordered NF request sets, the set of VNF types that the traffic passes through in order; $E_{j}^{NF}$ represents the logical link set, connecting the VNFs between the entrance and exit network element nodes; and $l_{j}$ represents the permissible end-to-end delay of the request. The CPU resource demand and memory resource demand of the u-th NF $v_{j,u}^{nf}\in V_{j}^{NF}$ of $sfc^{j}$ are $c_{j,u}^{nf}$ and $m_{j,u}^{nf}$, respectively. The traffic segment $\left(v_{j,u}^{nf},v_{j,v}^{nf}\right)$ of $sfc^{j}$ is represented as the logical link $e_{j,uv}^{nf}\in E_{j}^{NF}$, and the bandwidth demand of the logical link is $bw_{j,uv}^{nf}$.

The decision variable $t_{j,u}^{f}\in \left\{0,1\right\}$ specifies the types of NFs in the SFC. If the u-th virtual node $v_{j,u}^{nf}\in V_{j}^{NF}$ of $sfc^{j}$ is of type f, then $t_{j,u}^{f}$ equals 1:

(1) $$t_{j,u}^{f}=\{\begin{array}{c} 1,\;\;\;\;\;if\;v_{j,u}^{nf}is\;of\;type\;of\;f\in F\\ 0,\;\;\;\;\;\;\;\;\;\;\;\;\;\;\;\;\;\;\;\;\;\;\;\;\;\;\;\;\;\;\;\;otherwise \end{array}$$

It is stipulated that the entrance and exit network element nodes do not have a type; that is, $t_{j,u}^{f}$ is always equal to 0. When the decision variable $\eta_{j,u}^{i}\in \left\{0,1\right\}$ equals 1, it indicates that the u-th virtual node $v_{j,u}^{nf}\in V_{j}^{NF}$ of $sfc^{j}$ is deployed on the physical node $v_{i}^{s}\in V^{S}$:

(2) $$\eta_{j,u}^{i,t}=\{\begin{array}{c} 1,\;\;\;\;\;\;if\;v_{j,u}^{nf}is\;mapped\;to\;v_{i}^{s}\;during\;t\\ 0,\;\;\;\;\;\;\;\;\;\;\;\;\;\;\;\;\;\;\;\;\;\;\;\;\;\;\;\;\;\;\;\;\;\;\;\;\;\;\;\;\;\;\;otherwise \end{array}$$

When the decision variable $\tau_{j,uv}^{pq}\in \left\{0,1\right\}$ equals 1, it indicates that the traffic segment $e_{j,uv}^{nf}\in E_{j}^{NF}$ of $sfc^{j}$ flows through the physical link $e_{pq}^{S}\in E^{S}$, which can be represented as follows:

(3) $$\tau_{j,uv}^{pq}=\{\begin{array}{c} 1,\;\;\;\;\;\;\;if\;e_{j,uv}^{nf}is\;mapped\;to\;e_{pq}^{s}\;during\;t\\ 0,\;\;\;\;\;\;\;\;\;\;\;\;\;\;\;\;\;\;\;\;\;\;\;\;\;\;\;\;\;\;\;\;\;\;\;\;\;\;\;\;\;\;\;\;\;\;otherwise \end{array}$$

If an NF of type f∈F is mapped to $v_{i}^{s}\in V^{S}$, then a VNF of type f must also be instantiated on node $v_{i}^{s}$. Here we define the state variable $\beta_{f}^{i}\in \left\{0,1\right\}$, which equals 1 if there is a VNF of type f∈F on the physical node $v_{i}^{s}\in V^{S}$:

(4) $$\beta_{f}^{i}=\{\begin{array}{c} 1\;\;\;\;\;\;\;if\;a\;VNF\;of\;type\;f\;is\;assigned\;on\;v_{i}^{s}\\ 0\;\;\;\;\;\;\;\;\;\;\;\;\;\;\;\;\;\;\;\;\;\;\;\;\;\;\;\;\;\;\;\;\;\;\;\;\;\;\;\;\;\;\;\;\;\;\;\;\;\;\;\;\;\;otherwise \end{array},$$

(5) $$\beta_{f}^{i,t}=1,\;\mathrm{if}\;\underset{sfc^{j}\in S}{\sum }\underset{v_{j,u}^{nf}\in V_{j}^{NF}}{\sum }\eta_{j,u}^{i,t}t_{j,u}^{f}\geq 1,\;\forall v_{i}^{s}\in V^{S},\;f\in F,\;t\in T$$

Due to dynamic traffic changes, the relationship between the inflow bandwidth and resource demand of $v_{j,v}^{nf}\in V_{j}^{NF}$ at time t can be expressed as follows:

(6) $$c_{j,v}^{nf}\left(t\right)=bw_{j,uv}^{nf}\left(t\right)\underset{f\in F}{\sum }t_{j,v}^{f}\;{\mathrm{coeff}}_{c}^{f}\;\forall t\in T$$

(7) $$m_{j,v}^{nf}\left(t\right)=bw_{j,uv}^{nf}\left(t\right)\underset{f\in F}{\sum }t_{j,v}^{f}\;{\mathrm{coeff}}_{m}^{f}\;\forall t\in T$$

where $coeff_{c}^{f}$ and $coeff_{m}^{f}$ represent the CPU resource coefficient and memory resource coefficient, respectively, of the VNF of type f∈F. The data flow may be amplified or reduced (such as firewalls and intrusion defense will reduce traffic) when passing through each NF, causing the required bandwidth to change. The relationship between the inflow bandwidth of an NF $v_{j,v}^{nf}\in V_{j}^{NF}$ and the required bandwidth of the logical link connected to it is

(8) $$bw_{j,uv}^{nf}\left(t\right)=bw_{j,ku}^{nf}\left(t\right)\underset{f\in F}{\sum }t_{j,v}^{f}\;{\mathrm{ratio}}^{f}\;\forall t\in T$$

where $v_{k}^{nf},v_{u}^{nf},v_{v}^{nf}\in V_{i}^{NF}$ are a set of ordered NFs, and ${\mathrm{ratio}}^{f}$ is the bandwidth scaling factor for VNF of type f∈F.

To describe the migration process, here we define variables related to server status and VNF migration. When $\xi_{j,u}^{t}\in \left\{0,1\right\}$ equals 1, it indicates that the virtual node $v_{j,u}^{nf}$ needs to perform migration in the t period.

We define a set of variables related to resource prediction. Resource data can be CPU, disk, memory, or other system resource usage, such as the number of processes, OS load, etc. For simplicity, this paper considers predicting CPU and memory data. $c_{j,u}^{nf}\left(t+n\right)\left(n=1,2,\ldots \right)$ represents the CPU resource demand of $v_{j,u}^{nf}$ in the t+n period obtained through prediction; $m_{j,u}^{nf}\left(t+n\right)\left(n=1,2,\ldots \right)$ represents the memory resource demand of $v_{j,u}^{nf}$ in the t+n period obtained through prediction.

$thr_{over}$ represents the overload threshold of the underlying server.

The above variables satisfy the following constraint conditions: each virtual network function of the service function chain should be deployed on a physical node, which is unique, and can be represented as follows:

(9) $$\underset{v_{i}^{s}\in V^{s}}{\sum }\eta_{j,u}^{i,t}=1\;\forall sfc^{j}\in S,\;\forall v_{j,u}^{nf}\in V_{j}^{NF},\;\forall t\in T$$

Each virtual link needs to be mapped onto a physical link. Since the virtual link may span nodes,

(10) $$\underset{e_{pq}^{s}\in E^{s}}{\sum }\tau_{j,uv}^{pq,t}\geq 1\;\forall sfc^{j}\in S,\;\forall e_{j,uv}^{nf}\in E_{j}^{NF},\;\forall t\in T$$

The constraints on CPU and memory resources of the underlying network nodes are respectively satisfied:

(11) $$\underset{f\in F}{\sum }\beta_{f}^{i,t}c^{f}+\underset{sfc^{j}\in S}{\sum }\underset{v_{j,u}^{nf}\in V_{j}^{NF}}{\sum }\eta_{j,u}^{i,t}c_{j,u}^{nf}\left(t\right)\leq C_{i}^{S}\;\forall v_{i}^{s}\in V^{S},\;\forall t$$

(12) $$\underset{f\in F}{\sum }\beta_{f}^{i,t}m^{f}+\underset{sfc^{j}\in S}{\sum }\underset{v_{j,u}^{nf}\in V_{j}^{NF}}{\sum }\eta_{j,u}^{i,t}m_{j,u}^{nf}\left(t\right)\leq M_{i}^{S}\;\forall v_{i}^{s}\in V^{S},\;\forall t\in T$$

The underlying link bandwidth constraint is satisfied:

(13) $$\underset{sfc^{j}\in S}{\sum }\underset{e_{j,uv}^{nf}\in E_{j}^{NF}}{\sum }\tau_{j,uv}^{pq,t}bw_{j,uv}^{nf}\left(t\right)\leq BW_{pq}^{S}\;\forall e_{pq}^{s}\in E^{S},\;\forall t\in T$$

The service function chain delay limit is satisfied:

(14) $$\underset{e_{j,uv}^{nf}\in E_{j}^{NF}}{\sum }\underset{e_{pq}^{s}\in E^{s}}{\sum }\tau_{j,uv}^{pq,t}L_{pq}^{S}\leq l_{j}\;\forall sfc^{j}\in S,\;\forall t\in T$$

Every pair of connected VNFs $\left(v_{j,u}^{nf},v_{j,v}^{nf}\right)$ satisfies traffic conservation. Given a logical link $e_{j,uv}^{nf}\in E_{j}^{NF}$ and underlying nodes $\forall v_{i}^{s}\in V^{S}$,

(15) $$\sum_{v_{q}^{s}\in \Omega^{+}\left(v_{p}^{s}\right)}\tau_{j,uv}^{qp,t}-\sum_{v_{q}^{s}\in \Omega^{-}\left(v_{p}^{s}\right)}\tau_{j,uv}^{pq,t}=\left\{\begin{array}{c} \eta_{j,v}^{p,t}-\eta_{j,u^{\prime }}^{p,t},\;\;\;\;\;\;\;\;\;\;\;\;\;\;\;\;\;\;\;\;\;\;\;\;\forall v_{j,u^{\prime }}^{nf},v_{j,v}^{nf}\in V_{j}^{NF}\\ 1,\;\forall v_{j,v}^{nf}\in \{v_{j,out}^{s}\},\;v_{j,out}^{s}=v_{p}^{s},\;v_{j,u}^{nf}\in V_{j}^{NF}\\ -1,\;\forall v_{j,v}^{nf}\in \{v_{j,in}^{s}\},\;v_{j,in}^{s}=v_{p}^{s},\;v_{j,u}^{nf}\in V_{j}^{NF}\\ 0,\;\;\;\;\;\;\;\;\;\;\;\;\;\;\;\;\;\;\;\;\;\;\;\;\;\;\;\;\;\;\;\;\;\;\;\;\;\;\;\;\;\;\;\;\;\;\;\;\;\;\;\;\;\;\;otherwise \end{array}\right.$$

where $\Omega^{+}\left(v_{p}^{s}\right)$ and $\Omega^{-}\left(v_{p}^{s}\right)$ represent the upstream and downstream node sets of *vps*, respectively.

The migration of the virtual node $v_{j,u}^{nf}\in V_{j}^{NF}$ is represented by the following constraint condition. If the underlying node mapped by $v_{j,u}^{nf}$ is different before and after the traffic change, then $v_{j,u}^{nf}$ is migrated:

(16) $$\xi_{j,u}^{t}\geq \eta_{j,u}^{i,t}-\eta_{j,u}^{i+1,t}\;\forall v_{j,u}^{nf}\in V_{j}^{NF},\;\forall sfc^{j}\in S,\;\forall v_{i}^{s}\in V^{S},\;\forall t\in T$$

### 3.3. Load Balancing Model

The network system can more readily adjust to the dynamic fluctuations in network traffic because of network load balancing [3]. Load balancing is implemented for time-varying network traffic in order to improve network resilience following VNF migration and encourage long-term resource optimization.

The system’s efficiency in terms of load balancing is assessed using the change in resources. The average resource usage rate of physical nodes is directly correlated with the variance of system resources [12]. The load on physical node m may change as a result of VNF j being moved from physical node n to physical node m during the VNF migration process.

At this time, the load on physical node m can be represented as follows:

(17) $$L_{m}^{\mathrm{cpu}}\left(t\right)=\underset{j\in N_{i}^{v}}{\sum }x_{m}^{i,j}\cdot C_{i,j}^{v}\left(t\right)$$

(18) $$L_{m}^{\mathrm{mem}}\left(t\right)=\underset{j\in N_{i}^{v}}{\sum }x_{m}^{i,j}\cdot M_{i,j}^{v}\left(t\right)$$

where the CPU and memory resource loads of physical node m are denoted by $L_{m}^{\mathrm{cpu}}\left(t\right)$ and $L_{m}^{\mathrm{mem}}\left(t\right)$, respectively. To determine overall load balancing, the variation in resources on a single physical node m is insufficient. Based on variations in system resources, the load-balancing capability of the system can be assessed. Consequently, the mean CPU and memory load values at time t are:

(19) $$L_{\mathrm{mean}}^{\mathrm{cpu}}\left(t\right)=\frac{\sum_{m\in N^{P}}L_{m}^{\mathrm{cpu}}\left(t\right)/C_{m}}{\left|N^{P}\right|}$$

(20) $$L_{\mathrm{mean}}^{\mathrm{mem}}\left(t\right)=\frac{\sum_{m\in N^{P}}L_{m}^{\mathrm{mem}}\left(t\right)/M_{m}}{\left|N^{P}\right|}$$

where $C_{m}$ and $M_{m}$ represent the physical node m’s CPU and memory resource capacity, respectively. The network’s variations in CPU and memory resources are stated as follows:

(21) $$L_{\mathrm{var}}^{\mathrm{cpu}}\left(t\right)=\frac{\sum_{m\in N^{P}}{\left[L_{m}^{\mathrm{cpu}}\left(t\right)-L_{\mathrm{mean}}^{\mathrm{cpu}}\left(t\right)\right]}^{2}}{\left|N^{P}\right|}$$

(22) $$L_{\mathrm{var}}^{\mathrm{mem}}\left(t\right)=\frac{\sum_{m\in N^{P}}{\left[L_{m}^{\mathrm{mem}}\left(t\right)-L_{\mathrm{mean}}^{\mathrm{mem}}\left(t\right)\right]}^{2}}{\left|N^{P}\right|}$$

We employ the percentage of resource consumption as the measurement unit when assessing a network system’s load balancing to make sure that storage and processing resources are quantified in the same unit. Consequently, the weighted sum of the variations of computing and storage resources can be used to determine the load-balancing capability $L_{\mathrm{total}}\left(t\right)$ of the network system, which is expressed as follows:

(23) $$L_{\mathrm{total}}\left(t\right)=\omega_{1}L_{\mathrm{var}}^{\mathrm{cpu}}\left(t\right)+\omega_{2}L_{\mathrm{var}}^{\mathrm{mem}}\left(t\right),$$

where the weight factors for the impact of storage and computing resources on network load balancing are denoted by $\omega_{1}$ and $\omega_{2}$, respectively. Both resources are assumed to have the same effect on the network in this paper, with $\omega_{1}=\omega_{2}=0.5$.

Thus, minimizing the variation in computing and storage resources is the system’s optimal goal, as indicated by

(24) $$minL_{\mathrm{total}}\left(t\right)$$

## 4. Algorithm Design

Liu et al. [13] demonstrated that the VNF migration problem is NP-hard, since the significant scale of the network, the diversity of the network service flow, and the variability of the network environment make the VNF placement decision space high-dimensional and difficult.

**Figure 2:**
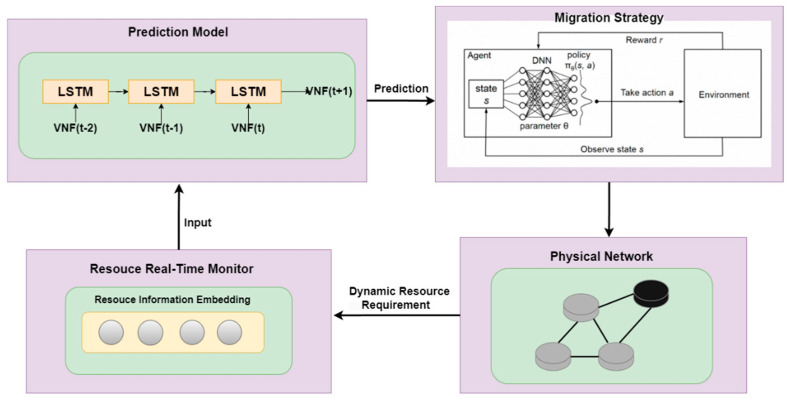
Overall framework of the RP-LBM.

Therefore, this section proposes a Resource Predictive Load Balancing SFC Migration Algorithm (RP-LBM) to address the aforementioned issue. The overall architecture of the algorithm is shown in Figure 2. The resource monitoring module obtains the dynamic resource utilization of the physical network. The Long Short-Term Memory (LSTM) model uses historical resource demand data to predict the NF resource demand at a future time. The resource prediction result is input into the migration decision module. For the SFC deployed on the node that is expected to be overloaded, the Proximal Policy Optimization algorithm is used to obtain the migration strategy. This strategy includes determining the migration path and VNF embedding nodes, ensuring that the service is not interrupted, and minimizing resource variance.

### 4.1. LSTM-Based Resource Demand Prediction Algorithm

Traffic variations in network environments are typically highly real-time and dynamic. Short-term forecasting can promptly reflect and quickly capture these changes. Deep learning models possess rapid learning and adaptation capabilities, enabling them to accurately complete prediction tasks and capture real-time traffic fluctuations within a short time frame. Therefore, we have adopted an integrated algorithm that combines Convolutional Neural Networks, Long Short-Term Memory networks, and attention mechanisms to accurately predict the resource demands of VNF [14].

First, the CNN module is used to extract key features from the historical data of VNF resource usage. CNNs excel at identifying local patterns and spatiotemporal features in data, and are able to generate high-quality feature representations. The convolutional layer scans the VNF resource usage time series data with multiple convolutional kernels. This captures different types of local patterns, such as peaks and regular changes in resource usage. As a result, it generates a rich set of feature maps that clearly show the underlying structure of VNF resource usage.

Next, the features extracted by the CNN are passed to the LSTM network. LSTM has powerful sequence processing capabilities, which can capture the time dependencies and long-term patterns in the use of VNF resources. This helps the model learn the connections between different time steps. LSTM processes the feature sequences and captures the dynamic information of resource demands change over time by maintaining hidden states and memory cells. LSTM can identify trend changes and cyclical fluctuations in resource use.

**Figure 3:**
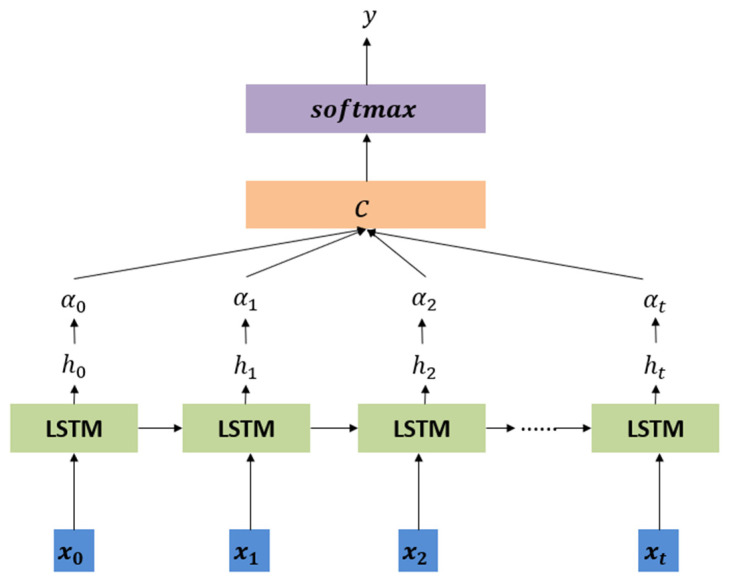
An LSTM model with attention.

To further improve the model’s prediction capability, we integrate an attention mechanism into the LSTM network. The attention mechanism can assign different weights to each time step. This highlights the most important historical information for the current prediction. Figure 3 shows a flowchart of how the attention mechanism is integrated with the LSTM network. The workflow of the attention mechanism is as follows:

At each time step t, the attention mechanism calculates the attention weights based on the hidden state of the current LSTM $h_{t}$ and the hidden state of all previous time steps $\alpha_{t,i}$, where i takes all previous time steps. Attention weights are calculated by a scoring function that measures the relevance of each hidden state $h_{i}$ to the current prediction task t:

(25) $$e_{t,i}=score\left(h_{t},h_{i}\right)=h_{t}^{⊺}W_{a}h_{i}$$

(26) $$\alpha_{t,i}=\frac{exp(e_{t,i})}{\sum_{k=1}^{T}exp(e_{t,k})}$$

where $W_{a}$ is a learnable weight matrix, and the softmax function ensures that the attention weights $\alpha_{t,i}$ are 1 on all i.

Then, the attention weight $\alpha_{t,i}$ is used to perform a weighted sum of the hidden states to generate a context vector $c_{t}$:

Finally, the context vector $c_{t}$ is $h_{t}$ connected to the current hidden state, and the final resource demand forecast ${\hat{y}}_{t}$ is generated through the fully connected layer:

(27) $${\hat{y}}_{t}=softmax\left(W_{c}\left[c_{t};h_{t}\right]+b_{c}\right)$$

where $W_{c}$ and $b_{c}$ are the weight matrix and bias term of the fully connected layer, respectively.

Through this hybrid model combining CNN, LSTM, and attention mechanisms, the paper achieves precise predictions of future VNF resource demands, providing a reliable basis for SFC migration.

### 4.2. PPO-Based SFC Migration Algorithm

In the study of VNF migration problems, traditional heuristic algorithms are widely used in practical scenarios due to their simplicity and low computational complexity. However, in high-dimensional and complex decision spaces, these algorithms tend to get stuck in local optima. This limits the overall effectiveness of migration strategy optimization.

Deep reinforcement learning (DRL) algorithms, with their self-learning capabilities and extensive data analysis power, can explore a more comprehensive strategy space in high-dimensional decision environments. This allows DRL algorithms to find superior migration strategies compared to heuristic algorithms in complex and dynamic network environments. Although training deep neural networks requires significant computational resources and time, they offer notable advantages in decision accuracy and migration strategy optimization for complex networks involving numerous network nodes, links, and service functions [4]. Additionally, the use of experience replay and parallel training techniques can improve training efficiency and reduce resource consumption.

Therefore, we propose a DRL algorithm based on Proximal Policy Optimization to solve the VNF migration problem. This algorithm converts the optimization objectives into a Markov Decision Process (MDP) and then uses the PPO algorithm to solve the MDP model, obtaining a near-optimal solution.

#### 4.2.1. MDP Model

As part of dynamic optimization, we take into account reducing the variance of network resources following VNF migration under time-varying traffic. Since the state space, action space, state transition probability, and reward function are represented by the four-element tuple  (S,A,P,R), respectively, the optimization aim is changed into a Discrete Time Markov Decision Process (DTMDP).

The network’s state space at time t is represented by the state space S, s(t)={ψ(t),ξ(t),C(t),M(t)}∈S, where ψ(t) denotes the state space of the physical node and ξ(t) denotes the state space of the physical link, respectively, as follows:

(28) $$\psi \left(t\right)=\left\{\varphi_{1}\left(t\right),\varphi_{2}\left(t\right),\ldots ,\varphi_{n}\left(t\right)\right\}\;\forall n\in N^{p}$$

(29) $$\xi \left(t\right)=\left\{S_{1,1}\left(t\right),S_{1,2}\left(t\right),\ldots ,S_{n,m}\left(t\right)\right\}\;\forall n,m\in N^{p},\;\forall l_{nm}\in L^{p}$$

The state space of the CPU and the memory resource requirements of VNF are represented by C(t) and M(t), respectively. These demand data reflect the anticipated outcome of VNF’s resource needs. The two resource requirements’ state spaces can be shown as follows:

(30) $$C\left(t\right)=\left\{C_{1,1}^{v}\left(t\right),C_{1,2}^{v}\left(t\right),\ldots ,C_{i,j}^{v}\left(t\right)\right\}\;\forall i\in F,j\in N_{i}^{v}$$

(31) $$M\left(t\right)=\left\{M_{1,1}^{v}\left(t\right),M_{1,2}^{v}\left(t\right),\ldots ,M_{i,j}^{v}\left(t\right)\right\}\;\forall i\in F,j\in N_{i}^{v}$$

The set of mapping activities that VNF can perform is denoted by action space A. The mapping action space of VNF can now be expressed as follows:

(32) $$a\left(t\right)=\left\{a_{1,1}\left(t\right),a_{1,2}\left(t\right),\ldots ,a_{i,j}\left(t\right)\right\}\;\forall i\in F,j\in N_{i}^{v},a\left(t\right)\in A$$

where $a_{i,j}\left(t\right)$ is the mapping action that the j-th VNF on SFC i may perform. This includes the selection scheme of the physical node mapping variable $x_{n}^{i,j}$ and the virtual link mapping variable $y_{n,m}^{j,k}$.

The transition probability P, which can be expressed as p(s(t+1)s(t),a(t)), is the probability of moving to the next state s(t+1) following an action a(t)∈a in the current state s(t)∈s.

The network creates a mapping strategy π when it performs action a(t) under state s(t). The optimization aims states that an instantaneous reward r(t) can be obtained if the mapping approach π fulfills system restrictions.

(33) $$r\left(t\right)=-L_{\mathrm{total}}\left(t\right)$$

The network uses the value function $V^{\pi }$ to assess the quality of the current VNF mapping strategy π at this precise instant. $V^{\pi }$ can be represented as follows:

(34) $$\begin{array}{ll} V^{\pi }\left(s\left(t\right),a\left(t\right)\right) & =E\left[R\left(t\right)s\left(t\right),a\left(t\right)\right]\\ & =E\left[r\left(t\right)+\gamma_{v}r\left(t+1\right)+\cdots s\left(t\right),a\left(t\right)\right]\\ & =E\left[r\left(t\right)+\gamma_{v}V^{\pi }\left(s\left(t+1\right),a\left(t+1\right)\right)s\left(t\right),a\left(t\right)\right] \end{array}$$

where the importance of future rewards in relation to present rewards is represented by the discount factor, $\gamma_{v}\in \left[0,1\right]$. The optimal strategy for VNF migration can be expressed as follows:

(35) $$\pi^{*}=\arg\underset{a}{\max}V^{\pi }\left(s,a\right)\;\forall s,a$$

#### 4.2.2. Proximal Policy Optimization Algorithm

Proximal Policy Optimization is a widely used policy optimization algorithm in reinforcement learning. PPO aims to stabilize the training process and reduce fluctuations and uncertainties during policy updates by limiting the difference between new and old policies [15]. PPO is mainly implemented through the following key steps.

First, it interacts with the environment using the current policy to collect a batch of trajectory data, including states $s_{t}$, actions $a_{t}$, rewards $r_{t}$, and next states $s_{t+1}$. To improve sample efficiency, data are usually collected simultaneously in multiple parallel environments. These data are used for subsequent updates of the policy and value function.

Next, the Generalized Advantage Estimation (GAE) method is used to estimate the advantage function $\hat{A_{t}}$. The advantage function measures the superiority of a particular action relative to the average policy:

(36) $$\hat{A_{t}}=\delta_{t}+\left(\gamma \lambda \right)\delta_{t+1}+\ldots +{\left(\gamma \lambda \right)}^{T-t+1}\delta_{T-1}$$

where $\delta_{t}=r_{t}+\gamma V\left(s_{t+1}\right)-V\left(s_{t}\right)$, γ is the discount factor, and λ is the GAE decay parameter.

Next, for each sample point, the probabilities of selecting action $a_{t}$ under state $s_{t}$ for both the new and old policies, $\pi_{\theta_{\mathrm{old}}}\left(a_{t}|s_{t}\right)$ and $\pi_{\theta }\left(a_{t}|s_{t}\right)$, are calculated. The probability ratio r(θ) indicates the relative change between the new and old policies. A ratio greater than 1 indicates that the new policy is more inclined to choose action $a_{t}$ in this state, while a ratio less than 1 indicates the opposite:

(37) $$r\left(\theta \right)=\pi_{\theta }\left(a_{t}|s_{t}\right)/\pi_{\theta_{\mathrm{old}}}\left(a_{t}|s_{t}\right)$$

To limit the extent of policy updates and avoid r(θ) being too large or too small, PPO introduces a clipping operation. The clipped objective function is defined as follows:

(38) $$L^{\mathrm{CLIP}}\left(\theta \right)=E_{t}\left[\min\left(r_{t}\left(\theta \right)\hat{A_{t}},\;\mathrm{clip}\left(r_{t}\left(\theta \right),1-\epsilon ,1+\epsilon \right)\hat{A_{t}}\right)\right]$$

where ϵ is a small positive number, typically between 0.1 and 0.3. The clipping operation constrains $r_{t}\left(\theta \right)$ within the range [1−ϵ, 1+ϵ], preventing excessive policy updates that lead to unstable training.

In addition, PPO uses mean squared error to minimize the difference between the value function and the target value:

(39) $$L^{\mathrm{VF}}\left(\theta \right)=E_{t}\left[{\left(V_{\theta }\left(s_{t}\right)-V_{t}^{\mathrm{target}}\right)}^{2}\right]$$

The value function $V_{\theta }\left(s_{t}\right)$ estimates the expected return after executing the policy in state $s_{t}$, while the target value $V_{t}^{\mathrm{target}}$ estimates the actual return, which can be calculated using Monte Carlo or temporal difference methods.

To encourage exploration and prevent premature convergence to local optima, an entropy regularization term is added. The entropy regularization term rewards policies with higher entropy, i.e., more random policies, which helps ensure adequate exploration during the early stages of training:

(40) $$S\left[\pi_{\theta }\right]\left(s_{t}\right)=-\underset{a}{\sum }\pi_{\theta }\left(a|s_{t}\right)\log\pi_{\theta }\left(a|s_{t}\right)$$

Combining these components, the total loss function L(θ) is defined as follows:

(41) $$L\left(\theta \right)=E_{t}\left(L^{\mathrm{CLIP}}\left(\theta \right)-c_{1}L^{\mathrm{VF}}\left(\theta \right)+c_{2}S_{\pi \theta }\right)$$

where $c_{1}$ and $c_{2}$ are weights used to balance the importance of policy loss, value function loss, and the entropy regularization term.

To optimize the total loss function L(θ), PPO typically uses stochastic gradient descent algorithms (such as the Adam optimizer) for parameter updates.

First, based on the current batch of sample data, calculate the gradients of the total loss function L(θ) with respect to the policy parameters and value function parameters, $\nabla_{\theta_{a}}L\left(\theta \right)$ and $\nabla_{\theta_{c}}L\left(\theta \right)$. Use the Adam optimizer to update the parameters based on the computed gradient information:

(42) $$\nabla_{\theta_{a}}L\left(\theta \right)\theta \leftarrow \theta -\alpha \cdot \frac{m_{t}}{\sqrt{v_{t}}+\epsilon }$$

where α is the learning rate; $m_{t}$ and $v_{t}$ are the first- and second-moment estimates of the gradient, respectively; and ϵ is a small constant to prevent division by zero.

The policy parameters $\theta_{a}$ aim to maximize policy rewards by minimizing the negative terms in the loss function:

(43) $$\theta_{a}\leftarrow \theta_{a}-\alpha \cdot \nabla_{\theta_{a}}\left(-L^{\mathrm{CLIP}}\left(\theta \right)-c_{2}S_{\pi_{\theta }}\right)$$

The value function parameters $\theta_{c}$ aim to minimize the value function loss $L^{\mathrm{VF}}\left(\theta \right)$:

(44) $$\theta_{c}\leftarrow \theta_{c}-\alpha \cdot \nabla_{\theta_{c}}L^{\mathrm{VF}}\left(\theta \right)$$

Through these steps, PPO effectively balances exploration and exploitation during policy optimization, stabilizes the training process, and enhances sample efficiency.

### 4.3. Resource Predictive Load Balancing SFC Migration Algorithm

The pseudocode for the RP-LBM algorithm is shown in Algorithm 1. RP-LBM uses predicted future resource demands and the physical network graph as inputs to generate the SFC mapping strategy π (Lines 1–2). First, it calculates the resource utilization $\eta_{R}$ for each physical node based on the prediction results (Line 3). If $\eta_{R}$ exceeds the set threshold $thr_{over}$ (Line 4), it selects an SFC to migrate on that node (Line 5) and it initializes the policy parameters $\left(\theta_{c},\;\theta_{a}\right)$, the maximum number of iterations $K_{max}$, and other relevant parameters (Line 6). In the main loop (Lines 7–19), it selects mapping actions a(t) from the policy $\pi \left(s_{n}\left(t\right)|a_{n}\left(t\right),\theta_{a}^{n}\right)$, executes the action to obtain an immediate reward *r*(t), and transitions to a new state *s*(t+1), while calculating the advantage function $A\left(s_{n}\left(t\right),a_{n}\left(t\right)\right)$. If constraints are not satisfied, it sets the immediate reward *r*(*t)* = −1/ε and reselects actions. Then, it computes the clipped surrogate objective for Proximal Policy Optimization and updates the policy parameters $\theta_{a}$ using gradient ascent and the value function parameters $\theta_{c}$ using gradient descent. If the resource utilization does not exceed the threshold, no action is taken (Line 20).
**Algorithm 1** Resource Predictive Load Balancing SFC Migration Algorithm (RP-LBM)1**Input:** Prediction result $c_{j,u}^{nf}\left(t+n\right),\;\;m_{j,u}^{nf}\left(t+n\right)\left(n=1,2,\ldots \right);$ Physical network diagram $G=\left(V^{s},E^{s}\right)$; SFC network diagram $G_{i}^{nf}=\left(V_{i}^{nf},\;E_{i}^{nf}\right)$2**Output:** SFC mapping strategy π;3Calculate the resource utilization $\eta_{R}$ of each physical node according to the prediction result;4if $\eta_{R}\;\geq \;thr_{over}$ then5  Select an SFC to migrate on that node;6  Initialize $(\theta_{c},\;\theta_{a}),K_{max}$, M, ($\left(\varepsilon_{c},\;\varepsilon_{a}\right)$);7  **for** episode = 1, …, M **do**8    Select mapping actions a(t) from the strategies $\pi \left(s_{n}\left(t\right)|a_{n}\left(t\right),\theta_{a}^{n}\right)$;9    **if** constraints are satisfied then10      Execute the action a(t), get the instantaneous reward11      *r*(t) and transfer it to the state *s*(t+1);12      Obtain the advantage function $A\left(s_{n}\left(t\right),a_{n}\left(t\right)\right)$;13    **else**14    Set instantaneous reward *r(t*) = −1/ε, and re-select the action a(t) from the policy network;15    **end if**16    Compute the clipped surrogate objective for PPO17    Update the policy parameters $\theta_{a}$ using gradient ascent18    Update the value function parameters $\theta_{c}$ using gradient descent19  **end for**20**end if**

## 5. Performance Evaluation

### 5.1. Simulation Setup

In order to evaluate the effectiveness of the above algorithm, we conducted simulations using the SFC simulation platform SFCSim [16] using the Python environment and tested the program on a cellular network topology.

**Figure 4:**
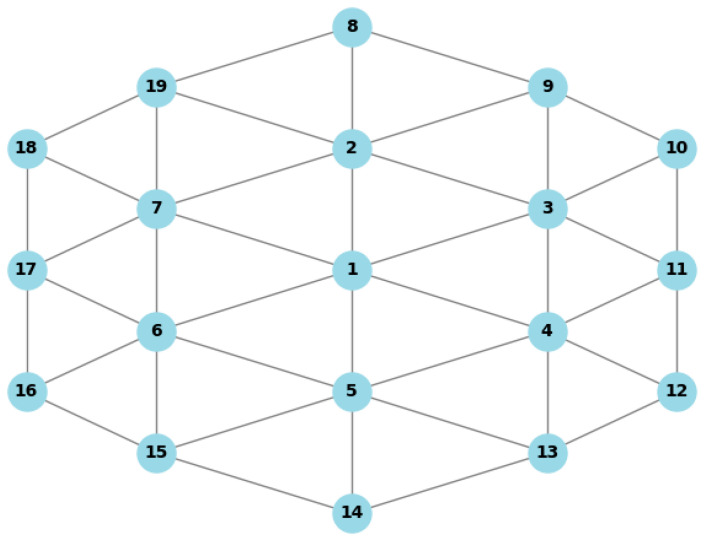
Cellular network topology.

Figure 4 depicts the physical network, which consists of 42 links and 19 physical nodes. The server’s CPU and memory resources are set to roughly (250, 300) MIPS and (600, 1000) GB, respectively, uniformly divided [17]. The link delay is uniformly distributed in units of (1, 4) ms, and we have set the link bandwidth capacity to 500 Mbps. The node overload threshold is 0.7.

We consider eight types of VNFs. Each SFC chooses a subset of VNFs at random, and the length of each SFC is randomly selected from two to five. The entrance and exit are chosen at random from $V^{S}$. The bandwidth factor ratios of the various VNF variants are chosen from 0.5, 1, and 1.5.

The CPU coefficient $coeff_{c}^{f}$ and memory coefficient $coeff_{m}^{f}$ of different types of VNFs are uniformly distributed in (0.1, 0.2). The maximum delay of the SFC is 30 ms. We set the network to have 20 paths in a 24-hour period using the Clearwater VNF Dataset’s traffic dataset [18].

### 5.2. Simulation Result and Analysis

In this paper, the following metrics are used to evaluate the performance of the algorithm under three conditions of sufficient resources, moderate resources, and scarce resources: node resource variance, total network migration times, average variance of network node resources, and service interruption rate, i.e., the ratio of interruption time to the SFC lifecycle.

Firstly, the performance of three algorithms in predicting the resource demand of each network function in the prediction network was compared: LSTM, CNN-AT-LSTM, and LSTM–encoder–decoder. The results of model training are shown in Table 1. It can be seen that the mean square error (MSE) and root mean square error (RMSE) between the predicted values and the actual values of the CNN-AT-LSTM model are the smallest, indicating that this model has the highest prediction accuracy.

**Figure 5:**
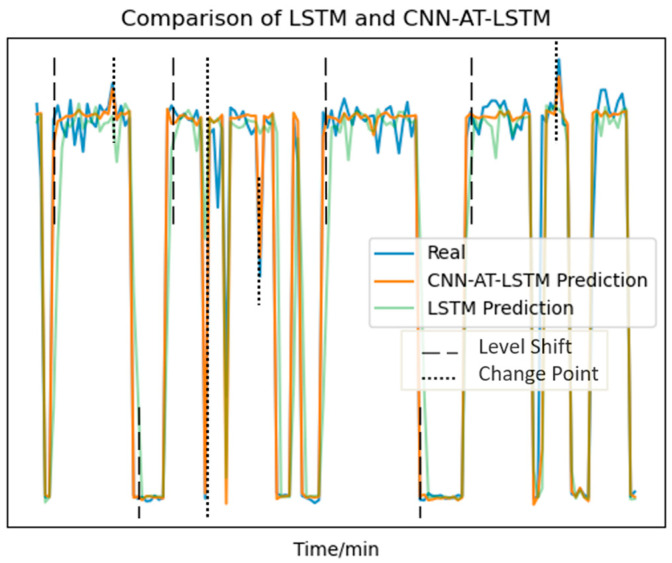
Comparison of model-predicted values and actual values.

To intuitively demonstrate the predictive capabilities of the models, we chose LSTM and CNN-AT-LSTM as examples. We compared the CPU demand predictions for a single VNF with the actual values. The detailed comparison results are presented in Figure 5, which includes annotations for “Level Shift” and “Change Point.” During times when the data experienced significant level shifts, CNN-AT-LSTM quickly adjusted its predictions and closely followed the changes in the actual values. In contrast, the LSTM model showed some delay. At data change points, the prediction errors of CNN-AT-LSTM were much smaller than those of the LSTM model. This highlights CNN-AT-LSTM’s ability to capture rapid dynamic changes more effectively. The results indicate that the attention mechanism in CNN-AT-LSTM allows it to focus on important time steps within the input sequence. This is especially useful during pattern shifts and data change points. As a result, CNN-AT-LSTM improves overall prediction accuracy and response speed. In summary, CNN-AT-LSTM offers higher prediction precision and reliability when dealing with the dynamic fluctuations of network resource demands. It is better equipped to handle severe and sudden variations in resource requirements compared to the LSTM model.

**Figure 6:**
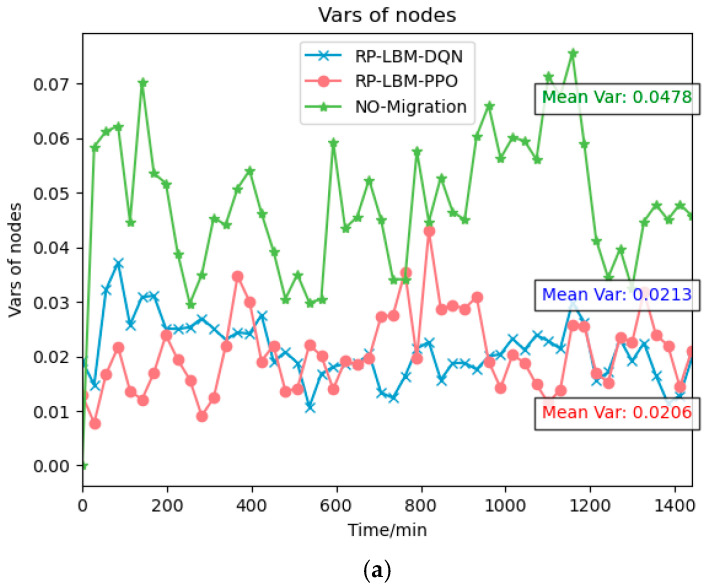

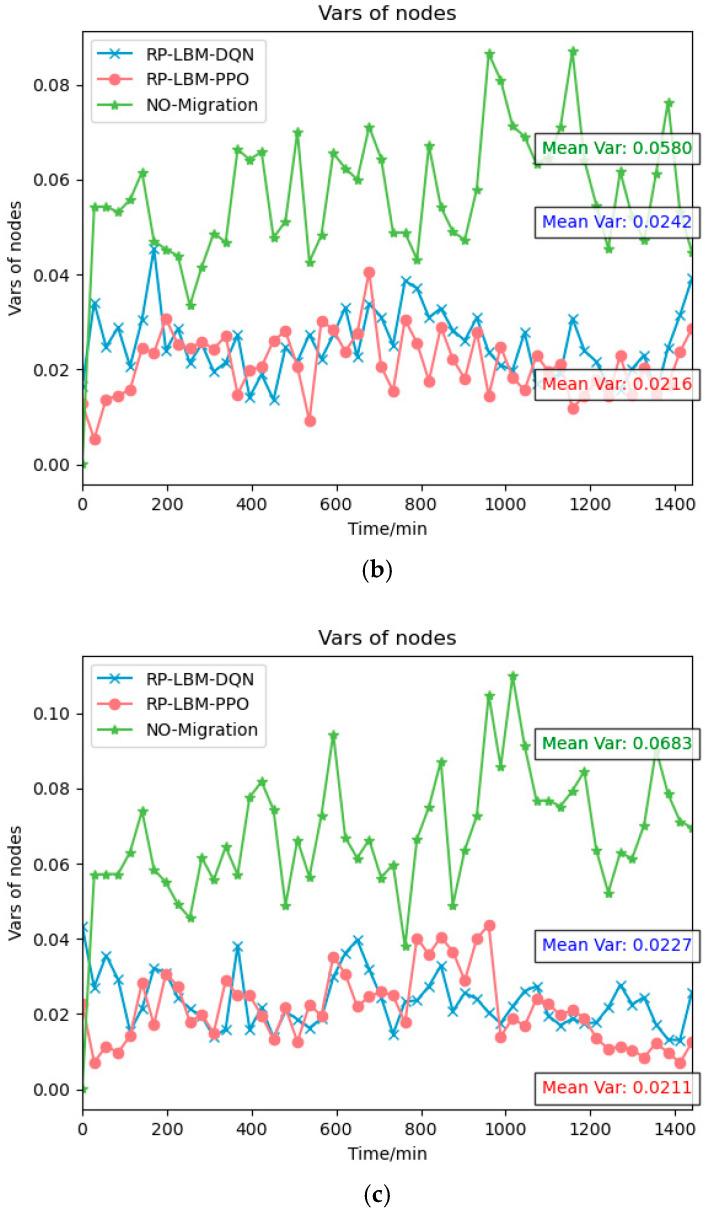
Comparison of network node resource variance when different algorithms are used under different resource conditions: (**a**) when resources are sufficient; (**b**) when resources are moderate; (**c**) when resources are scarce.

**Figure 7:**
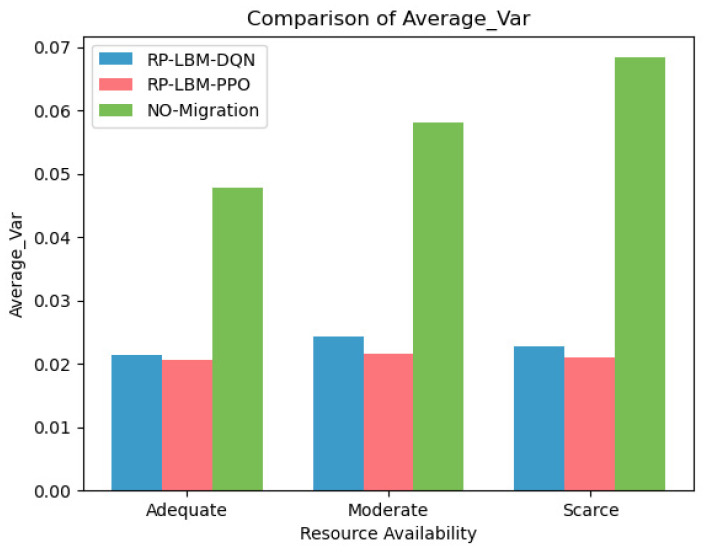
Comparison of average node resource variance under different resource conditions.

The resource demand prediction results serve as input for the RP-LBM. RP-LBM can identify potential overload nodes in advance and develop corresponding migration strategies. Figure 6 and Figure 7 illustrate the changes in network resource variance under three different resource states: (a) sufficient resources, (b) moderate resources, and (c) scarce resources. The figure also compares the average node resource variance under these different conditions. In each scenario, we use different deep reinforcement learning algorithms, namely DQN and PPO, to create migration strategies. We compare these strategies with situations where no pre-migration is performed. The reward functions of the DQN and PPO algorithms are based on the network system’s load-balancing capability. This design causes the algorithms to prefer migration strategies that minimize network resource variance. Consequently, both algorithms maintain a lower variance in network resource usage and support the network’s load-balancing state. In contrast, the no-migration strategy uses the shortest path algorithm to deploy SFC but does not optimize network node resource usage. This approach results in unbalanced network resource utilization and fails to manage resource allocation effectively. As a result, the overall performance and stability of the system may be negatively affected.

**Figure 8:**
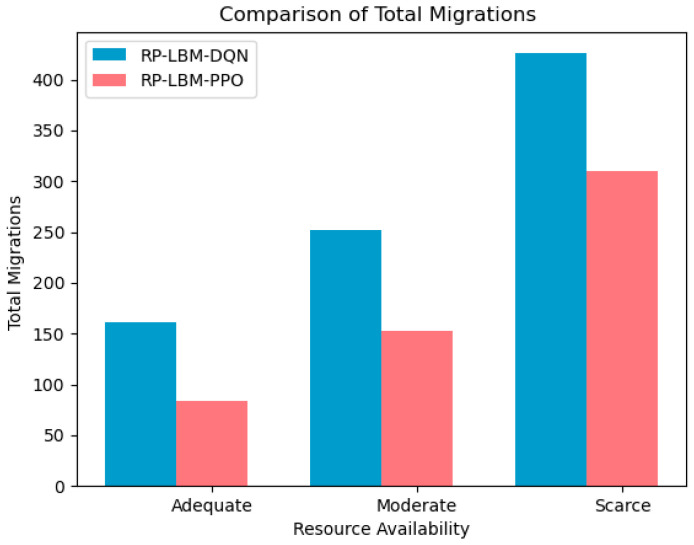
Comparison of total number of SFC migrations between RP-LBM-PPO and RP-LBM-DQN under different resource conditions.

**Figure 9:**
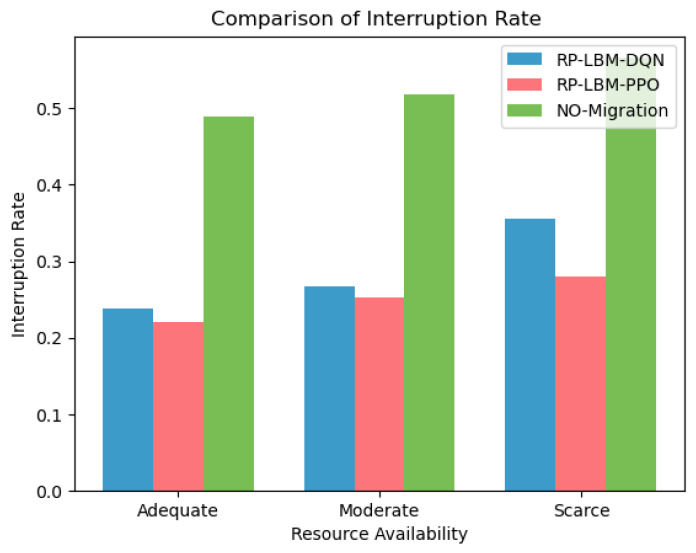
Comparison of service interruption rate between RP-LBM-PPO and RP-LBM-DQN under different resource conditions.

We also compare the total number of network SFC migrations and the SFC service interruption rates when using different algorithms, as shown in Figure 8 and Figure 9. The analysis shows that, across all three resource states, the RP-LBM-PPO algorithm always results in fewer SFC migrations than the RP-LBM-DQN algorithm. Additionally, when resources are sufficient or moderate, the PPO algorithm performs similarly to the DQN algorithm in terms of service interruption rates. This indicates that the PPO algorithm can maintain lower service interruption rates with less migration overhead. Figure 5 shows that resource demand has periodic fluctuations and sudden data surges. Throughout the traffic variations, the RP-LBM algorithm consistently maintains the service interruption rate low. This demonstrates the algorithm’s capability to quickly ensure service quality during traffic bursts. By rapidly adjusting resource allocation, the algorithm effectively maintains service continuity. These results further validate the superior performance of the RP-LBM-PPO algorithm in dynamic environments.

In contrast, the strategy that does not use pre-migration can only respond passively to changes in resource demand. This approach cannot develop a migration plan in advance and results in the highest service interruption rate. This finding highlights the importance of predicting resource demand and planning ahead to improve network stability and reduce the risk of interruptions. Combined with Figure 7, it can be known that when the network resource variance is small, resource utilization is more balanced. Balanced resource utilization directly impacts the overall performance and efficiency of the network. The PPO and DQN algorithms maintain lower levels of network resource variance through effective load-balancing strategies. In this way, these algorithms achieve more balanced resource allocation and reduce the service interruption rate.

However, when resources are scarce, the number of migrations using the DQN algorithm increases significantly. This leads to a sharp rise in the service interruption rate, which is 7.6% higher than that of the PPO algorithm. In a resource-scarce environment, the DQN algorithm tends to frequently adjust network configurations to balance the load. These excessive migration operations increase the system’s overhead and complexity, resulting in a higher service interruption rate. The PPO algorithm, on the other hand, uses a more stable policy optimization method. This approach allows the PPO algorithm to adjust policies more effectively under resource constraints, reducing unnecessary migration times. The policy update mechanism of PPO ensures the continuity and stability of network services with only small policy changes. Therefore, in situations where resources are limited, the PPO algorithm demonstrates greater efficiency and stability. It reduces the number of migrations while maintaining a low service interruption rate. This performance indicates that the PPO algorithm has better robustness and adaptability in complex network environments.

## 6. Conclusions

### 6.1. Summary of the Performance

To address the issue of the service QoS degradation or interruptions caused by dynamic changes in resource demand within edge computing environments, we conducted an in-depth study on the dynamic migration problem of SFCs where resources are scarce and constantly changing. The focus is on optimizing network performance through load balancing. We used the CNN-AT-LSTM machine learning model to predict the resource demands of VNFs. This approach allows us to anticipate changes in network node resource allocation and proactively develop migration strategies based on these predictions. By determining which VNFs to migrate from overloaded nodes, we ensure efficient resource management. We integrated the Proximal Policy Optimization deep reinforcement learning algorithm into our prediction model. This combination enables the system to detect changes in complex environments and select appropriate target nodes for migration. It also achieves network load balance, reduces the number of subsequent migrations, and minimizes the impact of unnecessary migrations.

The experimental results demonstrate that the RP-LBM-PPO algorithm outperforms the RP-LBM-DQN algorithm in both the number of migrations and the service interruption rate. When resources are sufficient or moderate, the PPO algorithm maintains a low service interruption rate with minimal migration overhead. However, in resource-scarce conditions, the DQN algorithm significantly increases the number of migrations and the service interruption rate due to frequent adjustments. In contrast, the PPO algorithm effectively reduces these negative impacts through stable policy optimization. These findings indicate that the PPO algorithm offers better efficiency and stability, making it more robust and adaptable in complex network environments.

### 6.2. Potential Future Research Directions

We assume that the lifecycle of SFC is infinite and do not consider how dynamic arrivals affect network load, migration operations, and service interruption rates. Additionally, the single-objective optimization algorithm we used focuses solely on minimizing the variance of system computation and storage resources to balance the network load. Although reducing the number of migrations and controlling service interruption rates have significantly improved the efficiency and stability of network resource management, we have not specifically modeled or deeply analyzed the time costs and resource overhead of migration operations. Future research should address SFCs with dynamic lifecycles and develop detailed models for migration costs and energy consumption [19]. Furthermore, it should explore multi-objective optimization methods to achieve a comprehensive optimization of resource utilization efficiency, service quality, and energy consumption.

## Figures and Tables

**Table 1:** Comparison of average results on all VNFs in SFCs between LSTM, CNN-AT-LSTM, and LSTM–encoder–decoder.

Metric	LSTM	CNN-AT-LSTM	LSTM–Encoder–Decoder
MSE	21.973	15.579	19.653
RMSE	4.688	3.947	4.433

## Data Availability

The original contributions presented in this study are included in the article. Further inquiries can be directed to the corresponding author.
